# Theoretical Formulation of Principal Components Analysis to Detect and Correct for Population Stratification

**DOI:** 10.1371/journal.pone.0012510

**Published:** 2010-09-17

**Authors:** Jianzhong Ma, Christopher I. Amos

**Affiliations:** Department of Epidemiology, The University of Texas M. D. Anderson Cancer Center, Houston, Texas, United States of America; University of Miami, United States of America

## Abstract

The Eigenstrat method, based on principal components analysis (PCA), is commonly used both to quantify population relationships in population genetics and to correct for population stratification in genome-wide association studies. However, it can be difficult to make appropriate inference about population relationships from the principal component (PC) scatter plot. Here, to better understand the working mechanism of the Eigenstrat method, we consider its theoretical or “population” formulation. The eigen-equation for samples from an arbitrary number (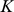) of populations is reduced to that of a matrix of dimension 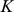, the elements of which are determined by the variance-covariance matrix for the random vector of the 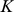 allele frequencies. Solving the reduced eigen-equation is numerically trivial and yields eigenvectors that are the axes of variation required for differentiating the populations. Using the reduced eigen-equation, we investigate the within-population fluctuations around the axes of variation on the PC scatter plot for simulated datasets. Specifically, we show that there exists an asymptotically stable pattern of the PC plot for large sample size. Our results provide theoretical guidance for interpreting the pattern of PC plot in terms of population relationships. For applications in genetic association tests, we demonstrate that, as a method of correcting for population stratification, regressing out the theoretical PCs corresponding to the axes of variation is equivalent to simply removing the population mean of allele counts and works as well as or better than the Eigenstrat method.

## Introduction

The genetic structure of populations is important both in population genetics and in genetic epidemiology. From the viewpoint of population genetics, detecting and quantifying population structure is crucial for understanding the demographic and evolutionary histories of populations [1], [2]. In genetic epidemiology, population stratification may induce false positives and must be corrected for [3], [4]. In both candidate gene association studies and genome-wide association studies (GWAS), unrecognized ancestral differences between the cases and controls are one of the main sources of spurious associations.

The most common methods used in the study of human population structure are clustering approaches [5]–[7] and principal components analysis (PCA) [1], [8], [9]. The most widely used clustering method, as implemented in the STRUCTURE program, provides the probability of group membership of samples [5]. This approach, however, is computationally intensive and hence is in practice not practical for analysis of large numbers of markers. Another problem with the clustering approach is that it assumes that the population of interest can be divided into distinct genetic groups, and therefore it is less suited to the situations where a subtle structure exists, or when there is association among individuals according to different attributes than the specified ancestries.

The PCA method was first applied to detecting and characterizing population structure more than 30 years ago [1]. By taking allele frequencies at different loci as a random vector and using the first few principal components (PCs), Cavalli-Sforza and co-workers constructed synthetic maps in their study of the evolutionary history of human populations [1], [2]. Recently, PCA has been applied to large-scale association studies using data for single-nucleotide polymorphisms (SNPs) in attempting to detect a few top axes of large genetic variation [10], [11].

In 2006, Patterson and co-workers [8] developed a new approach that uses PCA to detect population structures from large-scale genotype data of a sample of individuals. Instead of treating different markers as components and constructing PCs to represent the main variations from all markers as was traditional ([10] e.g.), in this new approach, Patterson et al. [8] indexed the random vector by individuals, taking genotype data at different markers as its realizations. In the resultant PC scatter plot using axes of the top PCs, individuals from different populations have different coordinates and thus have different locations. Price et al. [9] proposed a method of correcting for population stratification in association studies by regressing out the top PCs obtained by this new method from the genotype data. The method was implemented in the package EIGENSTRAT and is referred to as Eigenstrat method. The Eigenstrat method has been applied to quantifying fine structures and describing the relationships of many different populations, such as European American [12], [13], European [14], [15], and Japanese populations [16], and is now the gold standard for detecting and correcting for population stratification.

Although the Eigenstrat method is becoming more popular, appropriate inferences about the population relationships from the PC scatter plot remains a challenging task ([17] e.g.) In this paper, we address this issue by considering the theoretical or “population” formulation of the Eigenstrat PCA method. Here, the term “population” means that the PCA is formulated for a hypothetical marker with allele frequencies drawn from different distributions for different populations. In contrast, the term “sample” means that PCA is performed using markers observed on the sample. We establish an explicit connection between the pattern of the PC plot and the variance-covariance parameters of the random vector of allele frequencies. We propose that these parameters, independent of the relative sample sizes of the population, are more suitable than the patterns of PC plot for quantifying population divergence. Based on our theoretical formulation of PCA, we prove the existence of an asymptotic pattern of the PC plot when the population sizes become large, and derive the formula for numerically calculating this asymptotic pattern for given population parameters. We then illustrate how to apply our theory in quantifying population structures and relationships using HapMap [18] and simulated data. We also use the theoretical formulation to investigate the intra-population fluctuations on the PC scatter plot constructed using “sample” marker data.

Our theoretical formulation of PCA also applies to association studies. In the Eigenstrat method, the confounding effect of population structure is controlled for by regressing out the first few top sample PCs obtained from genotype data. Here, we propose that population stratification can also be corrected for by regressing out the theoretical or “population” PCs calculated from the estimates of the variance-covariance parameters using our formulation. This is not only an alternative to the Eigenstrat method in GWAS, but may also be applied in candidate gene association studies when fewer markers are available for study. It turns out, as we rigorously show, that this method is equivalent to subtracting the population mean of allele counts. The proposed method is tested and compared with other methods using simulations and found to have superior performance to the Eigenstrat method when the differentiation among populations was limited.

## Results

### Statistical model

Suppose we have genotype data for individuals sampled from 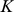 populations with sample sizes 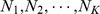, respectively. In Eigenfstrat theory, the data is modeled in the following way: the components of the *random* vector are the genotypes of the sampled individuals, 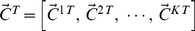 with 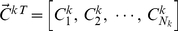. Here, 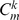 represents the count of the variant allele for individual 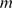 from population 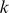 for a random marker. Data for different markers are taken as different measurements (“samples”) of this random vector. In this statistical model, the randomness of a component, 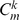, comes from two sources: the marker is randomly chosen, and the genotype is determined randomly conditional on the allele frequency of the chosen marker. The probability distribution of the allele frequency depends on which population the individual is from. The populations are characterized by the variance-covariance matrix of the random vector 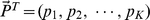 of allele frequencies
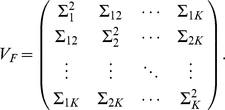
(1)


In this model, the genetically independent individuals are not statistically independent. Individuals from the same population have stronger correlations than those from different populations. We denote the variance-covariance matrix of the random vector 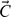 as
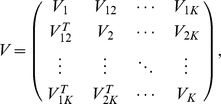
(2)where
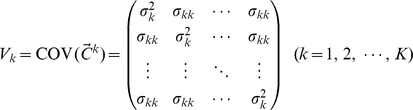
(3)and and
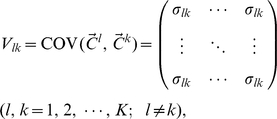
(4)where
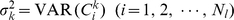
(5)

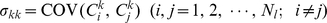
(6)

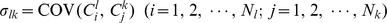
(7)are the variance of 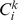 for any individual 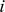 in population 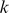, the covariance of two different individuals 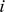 and 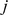 in population 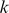, and the covariance of two individuals in populations 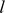 and 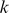, respectively. As shown in Text S1, 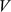 is related to the variance-covariance matrix of 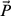 by
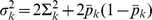
(8)

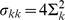
(9)

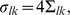
(10)where 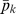 is the mean of 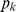, for 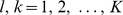 and 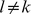.

### One-population case

For PCA, we need to calculate the eigenvalues and eigenvectors of the variance-covariance matrix of the random vector of interest [19]. We begin with the simplest case, where all individuals are from the same population. Useful insights can be gained from this trivial situation, as shown below. In this case, the variance-covariance matrix for the random vector 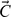 is given by Equation (3) with 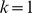, for which the eigensolutions can be easily obtained ([19] pp. 469–470). The eigenvalues can be divided into two groups. The first group includes only one large eigenvalue, 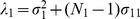, with the associated eigenvector 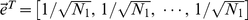. The second group includes all the other eigenvalues, which are smaller and are all equal: 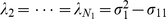. The coordinate of the random vector 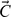 along the first PC is 
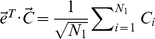
, proportional to the average of the allele counts over all samples. If the individuals within this population are not correlated to each other, 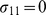 and 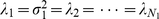. That is, 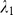 reduces to the small eigenvalue. In contrast, if the individuals are completely correlated (e.g. if all individuals are monozygotic twins) 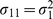, 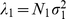, and 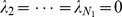. In general, the stronger the correlation between individuals, the larger 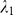 is and the smaller the small eigenvalues are. This means that the only PC here represents the co-variation of all individuals, whereas the small eigenvalues represent the variation between individuals.

Since there is only one population, the only PC with the large eigenvalue here does not reflect the variation caused by population structure. In Eigenstrat theory, therefore, one needs to perform the following mean adjustment:
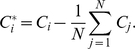
(11)For the mean-adjusted random vector 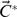, the variance-covariance matrix has the same structure as that of 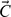, but with different diagonal and off-diagonal elements given by
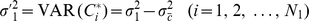
(12)

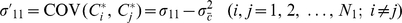
(13)

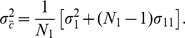
(14)It is easy to show that the large eigenvalue is now reduced to 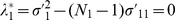 and the small eigenvalues remain unchanged:
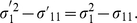
(15)This shows how the mean adjustment in Eigenstrat theory removes the overall variance represented by the first PC that reflects the joint variation of all components because of their correlation instead of stratification. The same mean-adjustment will be performed for the general case where individuals are from two or more populations, for the same reason.

### Two-population case

Now we turn to the first nontrivial case, where there are 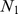 individuals in population 1 and 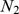 individuals in population 2 in the samples. In this case, the variance-covariance matrix of 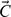 is
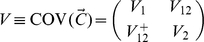
(16)and can be shown to have solutions as follows. The small eigenvalues of 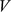 are just those of 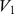 and 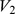, the same as in the one-population case. There are two large eigenvalues, each of which corresponds to an eigenvector whose coordinates are constant for individuals from the same population. However, these two large eigenvalues do not reflect only the variations caused by the population structure; as shown in the last subsection, they also represent the co-variation of the individuals. This can be easily seen if we assume 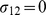. In this case, the two large eigenvalues are simply the large eigenvalues of 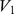 and 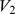. If we were only interested in detecting population structure, the PCA for 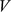 would be sufficient. However, we are also interested in correcting for the population stratification, and therefore we hope to obtain PCs mainly representing variations due to population structure. This is why we need to investigate the mean-adjusted vector 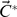 as defined in the last subsection. Here, again, the variance-covariance matrix of 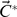 has the same structure as that of 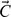 and has the following eigensolution. The small eigenvalues of 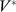 are still the same as those of 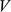. (Note that, as in the case of one population, 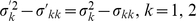.) The two large eigenvalues become 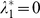 with eigenvector 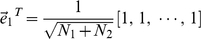 and
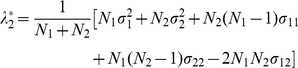
(17)with eigenvector
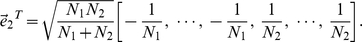
(18)Note that Equation (18) is equivalent to Equations (13a) and (16b) in [17]. The first large eigenvalue (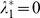) reflects the fact that the mean of the vector is zero, a result of the mean adjustment, whereas the second large eigenvalue represents the variation caused by the population structure. The only nonzero large eigenvalue (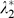) is very large compared with the small eigenvalues, for large 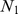 and 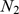. So, if there are only two populations, we would have only one eigenvector showing a clustering structure on a PC scatter plot using data of “samples” of markers. Any other eigenvectors would not have anything to do with stratification among populations.

It should be noted that the eigenvector corresponding to the only large eigenvalue and reflecting the population structure (Equation (18)) depends only on the ratio of sample sizes of the two populations, 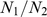, not on the other parameters (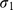, 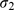, 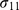, 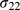, or 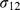). This is true only in the two-population case and is not a generic property, as will be clear soon.

### 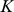-population case

In the general case of 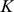 populations, the eigensolutions of the variance-covariance matrix Equation (2) are as follows. The small eigenvalues are the same as those for the individual populations. There are 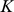 large eigenvalues, each corresponding to an eigenvector whose coordinates are constant for individuals from the same population. These large eigenvalues, again, reflect not only the variation caused by population stratification but also the overall co-variation of the individuals. After the mean adjustment, the variance-covariance matrix 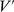 has the same structure as 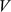 in Equation (2), and its submatrices 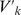 and 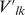 have the same structure as the corresponding 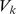 and 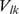 in Equations (3) and (4), respectively. The elements of 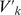 and 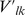 are given by
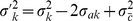
(19)

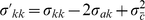
(20)

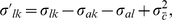
(21)where
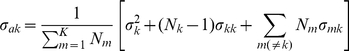
(22)

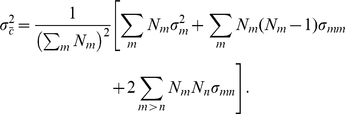
(23)


It is not difficult to show that the eigensolutions with small eigenvalues are still the same as those in the one-population and two-population cases (see also [8]). To find the eigensolutions with large eigenvalues of 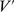, which describe the population distinctions, we define
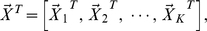
(24)and for each of the 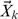 with dimension 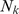,
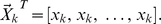
(25)Then the eigenequation
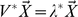
(26)is reduced to
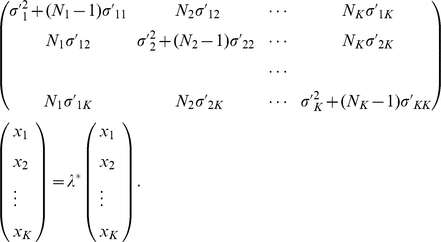
(27)By using the following identity, which is proven in Text S2,
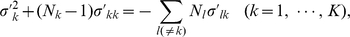
(28)the trivial eigensolution, reflecting the fact that the components of 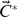 have a zero sum after the mean adjustment, is immediately obtained:
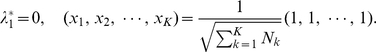
(29)This identity, (28), also means that any other nontrivial solutions must satisfy
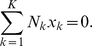
(30)The eigenvectors are usually normalized by
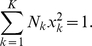
(31)Equation (27) can be used to numerically calculate the 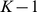 nontrivial eigenvalues and the corresponding eigenvectors for given variance-covariance parameters and sample sizes (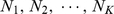). Thus, it provides a theoretical tool for connecting the patterns of PC scatter plot and the relationships between populations.

### Application to population genetics

#### PC plot patterns and population structure

The formulation we derived here not only provides a means of connecting PC plot patterns and population structure but also suggests an alternative to the 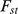 statistic for describing population relationships. The complete set of parameters describing the population relationships for 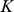 populations can be put into a vector of variance
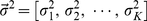
(32)and a covariance matrix
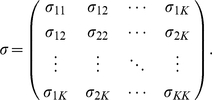
(33)These parameters, referred to as variance-covariance parameters, together with the sample sizes (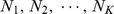), completely determine, by Equation (27), the theoretical (or “population”) eigenvectors. These eigenvectors, referred to as *axes of variation* in [8], are uniform within a population without a structure. Thus, when they are used to make a PC plot, each population is represented by a single point, which is referred to as the representative point of the population. Here, we illustrate, by using examples, how the pattern of the representative points can be used to infer population relationships.

**Figure 1:**
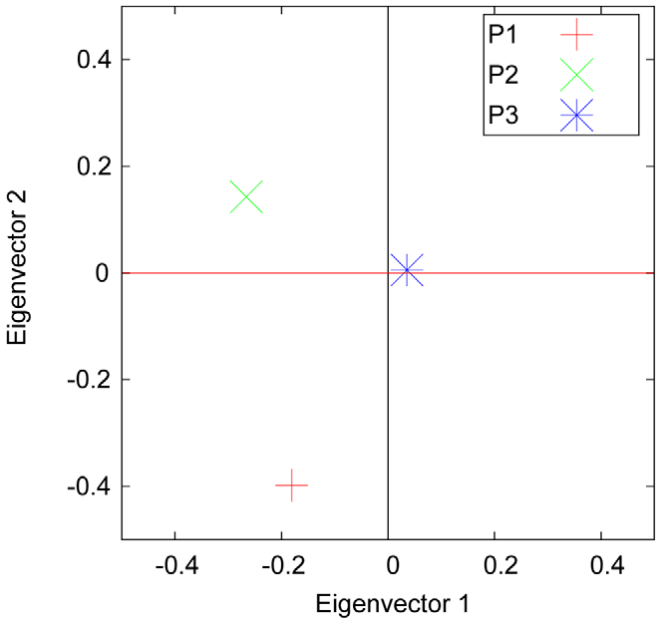
Example 1 of axes of variation calculated from variance-covariance parameters and sample sizes. The eigenvectors for the samples from three hypothetical populations, defined in Table 1, were calculated using Equation (27). Because the variance, within-population covariance and between-population covariance are all the same, the pattern shown here reflects purely the effect of sample sizes. Populations with small sample sizes are far away from the center, whereas populations with large sample sizes are around the center, as predicted by Equation (30).

In the first example, we illustrate the effect of population sizes on the pattern of a PC scatter plot. Although the variance and covariance between each pair of populations are the same, as shown in Table 1, the three representative points in the PC scatter plot are distributed unevenly because of the unbalanced sample sizes, as seen in Figure 1. The representative points of populations with small sample sizes are around the borders, whereas the points for populations with large sample sizes are located near the zero point, as can be explained by Equation (30).

**Table 1:** Parameters for the three populations in Figure 1.

	P1	P2	P3
Sample size^a^	5	10	100
Variance	1	1	1
Covariance			
P1	0.8	0.1	0.1
P2		0.8	0.1
P3			0.8

a Sample size: The number of individuals of each population.

**Figure 2:**
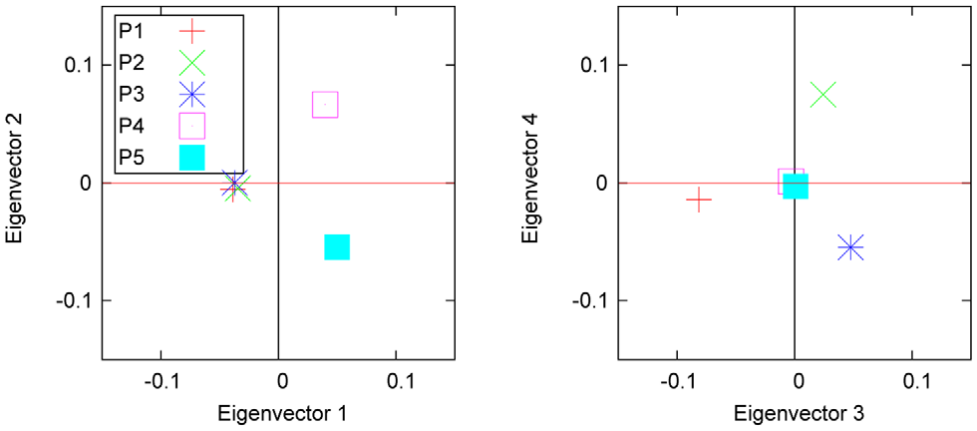
Example 2 of axes of variation calculated from variance-covariance parameters and sample sizes. The eigenvectors for the samples from five hypothetical populations, defined in Table 2, were calculated using Equation (27). Because the first three populations (P1, P2 and P3) are close to one another (the between-population covariance, 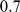, is only slightly smaller than the within-population covariances, 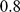, 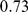 and 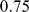, as shown in Table 2), they appear at the same location and are contrasted as a whole with the other two populations (P4 and P5) on the two-dimensional plot of the first two eigenvectors (left panel). P4 and P5 are contrasted in eigenvectors 2. The three closely related populations (P1, P2 and P3) are differentiated in eigenvectors 3 and eigenvectors 4.

In the second example, we have five populations, the first three of which are close to one another (in the sense that the corresponding covariances are large) and are far away from the other two distant populations (see Table 2). Figure 2 shows that the first three populations can hardly be distinguished from the two-dimensional PC plot using the first two eigenvectors. In eigenvector 1, P4 and P5 are contrasted with the three closely related populations (P1, P2 and P3), while in eigenvector 2 P4 is contrasted with P5, and the other three are in the middle. The three closely related populations are distinguished in eigenvectors 3 and 4. This example suggests that one has to examine a large enough number of eigenvectors in order to find all the significant population differences. The first two eigenvectors are the most important, but the others are also needed if the samples are from more than three populations. However, if there are only two populations, a two-dimensional PC plot is not needed; only the first eigenvector shows the population structure.

**Table 2:** Parameters for the five populations in Figure 2.

	P1	P2	P3	P4	P5
Sample size^a^	100	110	120	130	140
Variance	1	1	1	1	1
Covariance					
P1	0.8	0.7	0.7	0.1	0.11
P2		0.73	0.7	0.13	0.14
P3			0.75	0.14	0.09
P4				0.9	0.17
P5					0.91

a Sample size: The number of individuals of each population.

The representative points depend on the sample sizes as well as the variance-covariance parameters, as pointed out in [8]. We note that, even if equal sample sizes are used, the representative points cannot replace the variance-covariance parameters in characterizing the population relationships, because their values depend on the presence of one another in the analysis.

### Estimation of variance-covariance parameters and axes of variation

In practice, the variance-covariance parameters in Equations (32) and (33) are unknown and can only be estimated from genotype data of a large number of markers. The estimates of these parameters can be used to calculate the estimates of axes of variation using Equation (27). If the population memberships of the samples are known, estimation of the variance-covariance parameters is straightforward. For any one of the parameters in Equations (32) and (33), we can simply take the average of the corresponding elements of the sample variance-covariance matrix as its estimate. However, in reality, the information on population membership is usually unavailable and needs to be inferred using the PCA or other methods such as STRUCTURE. For inference of population structure from a PC plot, a generic clustering algorithm may be appropriate [20].

**Figure 3:**
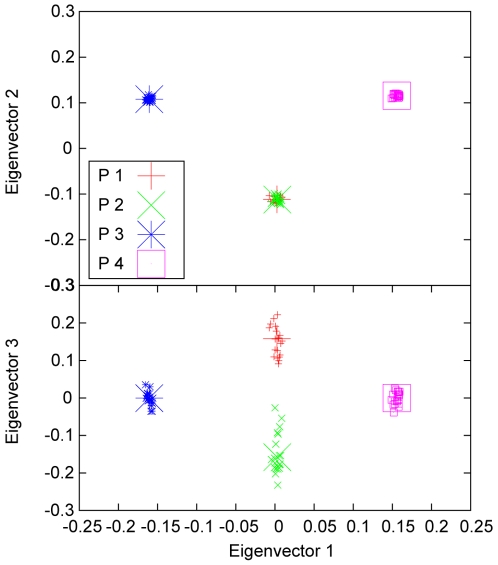
PC scatter plot and estimated axes of variation for a simulation. We plot the first three sample eigenvectors (small symbols) and the estimated axes of variation (large symbols) for a simulated data set with four populations with parameters given in Table 3. The three representative points were located in the centers of the clusters of the corresponding sample eigenvectors. The first two populations were contrasted only in eigenvector 3 because they were simulated using small 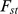 values and thus were close to each other.

In contrast, PCA in practice is based on the “sample” of markers. Namely, eigenvectors are calculated using genotypes of a large number of markers. These eigenvectors are therefore referred to as “sample” eigenvectors. The points on the PC scatter plot using the sample eigenvectors, called the sample points, are scattered to some extent because of sampling fluctuation. Our representative points using the estimated axes of variation should be located in the middle of the corresponding clusters of the sample points. We performed simulations to show how the axes of variation can be evaluated and to compare them with the sample eigenvectors used in Eigenstrat theory. Figure 3 shows an example of our simulation, using the simulating parameter values given in Table 3. P1 and P2 were simulated using an 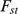 of 0.01 and thus were closer to each other than to P3 or P4, for which 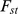 was much larger (0.43). The distance between P3 and P4 was even larger than their distances to P1 or P2. The representative points obtained from the estimated parameters listed in Table 3 were right in the centers of the corresponding sample points. Also listed in Table 3 are the estimated correlation coefficients 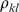 (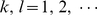). Compared with the covariances 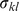, the corresponding correlations 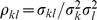 seem to be more suitable for representing the population distances.

**Table 3:** Simulating values for 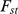 used for the four populations P1, P2, P3, and P4 and the estimated parameters.

	P1	P2	P3	P4
Sample size^a^	20	20	20	20
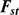	0.01	0.01	0.43	0.43
Variance	0.565	0.562	0.750	0.747
Covariance				
P1	0.128	0.119	0.114	0.116
P2		0.127	0.112	0.115
P3			0.500	0.113
P4				0.493
Correlation ( 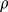 )				
P1	0.227	0.211	0.174	0.178
P2		0.225	0.172	0.178
P3			0.667	0.151
P4				0.660

a Sample size: The number of individuals of each population.

### Fluctuations in sample eigenvectors within populations and asymptotic PC plot patters

In practice, within-population fluctuations of the PC scatter plot using sample eigenvectors may be so strong that closely related populations have overlapping clusters and hence cannot be distinguished. Here, we first investigate the factors that affect the within-population fluctuations for a given population divergence: the sample sizes of the populations and the number of markers. We then study the asymptotic behavior of the patterns of the PC scatter plot as the sample size becomes large.

A remark is in order. The fluctuations of the sample points on the PC scatter plot within a population should not be confused with the random variation of marker data between individuals within a population. Recall from our theoretical consideration in Section 2 that the between-individual variation within a pure population exists even theoretically and is represented by the small eigenvalues and the corresponding eigenvectors. However, the within-population fluctuation of the sample points is mainly due to the limited “sample size” of markers and should be decreased as more markers are included in the analysis. The observed fluctuations of sample points around the representative points may also reflect effects from subtle population structure and cannot be reduced by increasing marker numbers.

**Figure 4:**
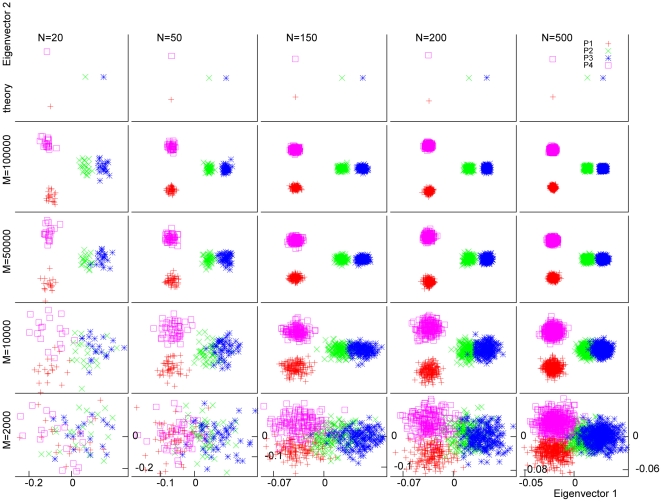
Effects of sample size and marker number on within-population fluctuations of PC scatter plot: eigenvector 2 vs. eigenvector 1. We plot the first two axes of variation (top row) and sample eigenvectors (other rows) for simulated data sets with different values for the number of markers (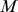) and the number of individuals (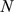) in the samples. In the simulation, we first generated three populations, P1, P2 and P4, each with the same 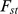 value of 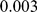. These three populations should therefore be equidistant from one another. Then another population, P3, was so generated that it was very close to P2 (see main text for details). P2+P3 can be viewed as a single population with a subtle structure. As 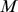 or 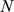 increased, the fluctuations within each population gradually decreased, while the distance between P2 and P3 remained unchanged.

**Figure 5:**
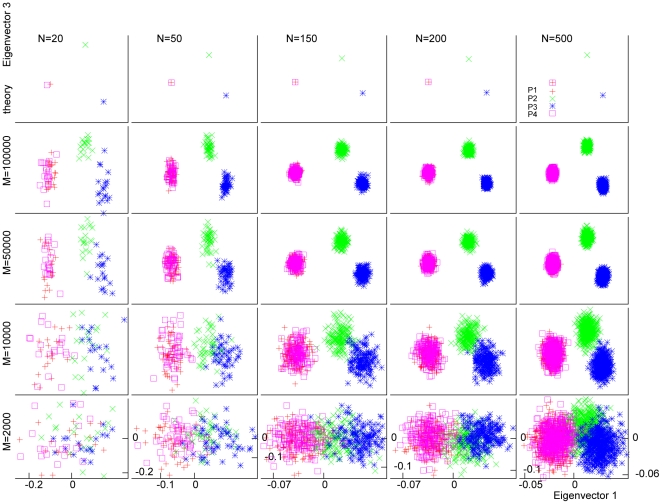
Effects of sample size and marker number on within-population fluctuations of PC scatter plot: eigenvector 3 vs. eigenvector 1. We plot the first and the third axes of variation (top row) and sample eigenvectors (other rows) for the same simulated data sets as in Figure 4. Since there were actually four populations, eigenvector 3 was needed to fully address the population differentiations. Indeed, we can see difference between P2 and P3 in eigenvector 3, in addition to that seen in eigenvector 1.

We performed simulations to demonstrate the effect of increasing population size (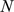) and number of markers (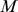) on the within-population fluctuations of sample points on the PC scatter plot. In our simulations, we first generated three populations (P1, P2, and P4) each with the same 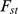 value of 0.003; the three populations should therefore be equidistant from one another. In order to mimic a subtle subpopulation structure, we created another population (P3) based on P2 by using the allele frequency vector of P2 added to a random vector, each of the elements of which was independently and uniformly distributed within 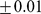. This resulted in three distinct populations, P1, P2+P3 and P4; P2+P3 has a subtle structure. Figure 4 shows the PC scatter plot using the first two eigenvectors for various values of 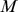 and 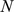. If the subtle structure within P2+P3 is ignored, this plot should be all that is needed to distinguish these three populations. As we see from this figure, as the number of markers (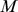) increased, the fluctuations within each population gradually decreased, but the distance between P2 and P3 remained unchanged, indicating that fluctuations due to limited “sample size” can be reduced by increasing it, whereas fluctuations reflecting subtle structure cannot. Since there were actually four populations, we also plotted the first and third eigenvectors in Figure 5. Here, we see that after mainly addressing the difference between P1+P4 and P2+P3 in eigenvector 1, and the difference between P1 and P4 in eigenvector 2, the difference between P2 and P3 was further addressed in eigenvector 3.

Figures 4 and 5 also show the effect of increasing the number of individuals in each population. As 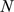 increased, the within-population fluctuations became smaller and the distinctions among populations became clearer. The effect of increasing 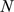 was much stronger than that of increasing 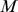, in agreement with what was found in [8]. Here, we give an explanation for this phenomenon as follows. For a finite number of markers, each individual in PCA actually acts as a population. When the number of individuals (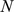) is small, the variation between individuals from the same population is comparable to that caused by population difference, so PCA tends to address these variations in the first few eigenvectors. As 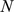 increases, the variation due to population differences becomes overwhelming, so PCA addresses only this variation in the first few eigenvectors and leaves the trivial ones to other eigenvectors with small eigenvalues.

In Figures 4 and 5, we also plotted the theoretical patterns calculated using the population sizes (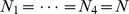) and the estimated variance-covariance parameters from the case with the largest 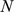 (500) and largest 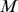 (100,000). The absolute distances between the representative points became smaller as 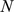 increased, because of the normalization equation (31), but the relative pattern remained almost the same, especially for large 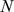. The ratio of distance between P2 and P3 to that between P2+P3 and P3 on the PC plot approached a constant value, implying that the pattern on the PC plot reached an asymptotic shape as 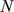 became large. This kind of asymptotic behavior can be derived from our theoretical considerations as follows.

**Figure 6:**
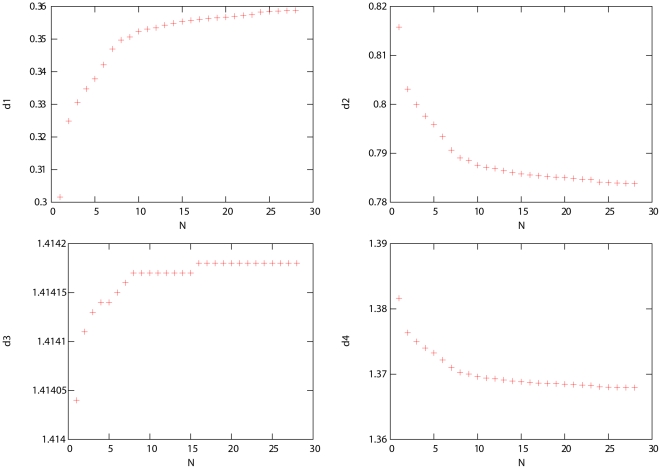
Approach to the asymptotic form. We plot the theoretical predictions for the dimensions of the PC plot pattern a function of the sample size for the simulated data sets used in Figures 4 and 5. Here, 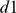 is the distance between P2 and P3 on eigenvector 1; 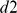 is the distance between P1 and P3 on eigenvector 1; 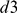 is the distance between P1 and P4 on eigenvector 2; and 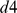 is the distance between P2 and P3 on eigenvector 3. When 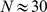, the pattern of the PC plot reached its asymptotic form.

Let 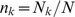 (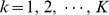) denote the relative sample size (or proportion) of population 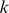 in the samples of interest. It is shown in Text S2 that the asymptotic form of the eigen-equation (27) is
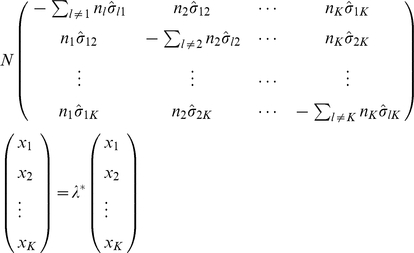
(34)with
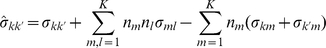
(35)for 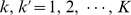 and 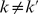. The small part being neglected for large 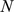 is
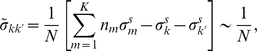
(36)where 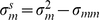 is the small eigenvalue for population 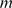. From Equation (34), we see that asymptotically the eigenvectors are independent of 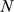 for given proportions of populations and variance-covariance parameters listed in Equations (32) and (33), whereas the large eigenvalues increase linearly with 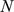. Figure 6 shows how the theoretical predictions of the dimensions of the pattern on the PC plot vary with sample size for the simulated datasets plotted in Figures 4 and 5. The dimensions shown are: 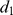, the distance between P2 and P3 on eigenvector 1; 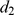, the distance between P1 and P3 on eigenvector 1; 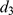, the distance between P1 and P4 on eigenvector 2; and 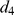, the distance between P2 and P3 on eigenvector 3. Here, the estimated variance-covariance parameters from the case with the largest number of markers (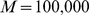) were used.

Note that the asymptotic form of the eigen-equation, and hence the asymptotic form of the PC plot patterns, do not depend on the values of the variances 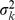 (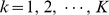). They are determined only by the intra- and inter-population covariances, and the relative sample sizes.

For 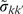 to be neglectable compared to 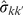, we need to have an 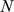 such that
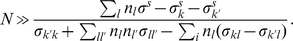
(37)In the simplest case, where all populations have the same variance (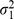) and the same intra-population covariance (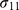) and each pair of populations has the same inter-population covariance (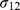), we have a simple expression for the critical size 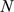 as
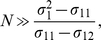
(38)indicating that the closer the populations (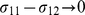) the larger the sample size needed for the asymptotic pattern to be approached. Note, however, that this is true only for cases where there are more than two populations in the sample. If there are only two populations, as shown in the previous section, the pattern in eigenvector 1 is determined only by the relative sample sizes (see Equation (18)).

### Application to HapMap data

**Figure 7:**
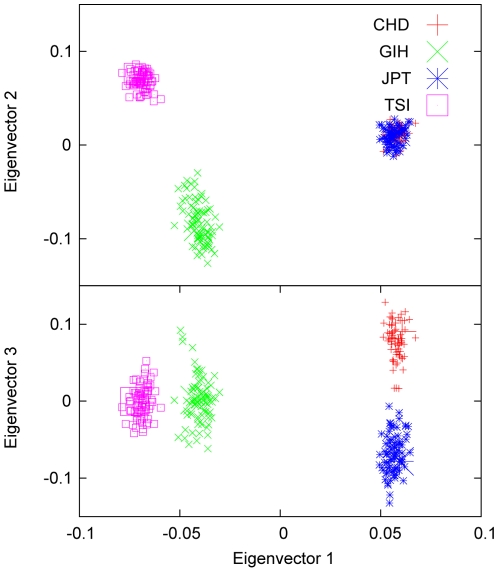
Four HapMap populations. The first three eigenvectors for the four populations CHD, GIH, JPT and TSI using the HapMap data for chromosome 1. As in Figure 3, the small symbols represent sample eigenvectors from the Eigenstrat analysis, whereas the large ones are the estimates of the axes of variation based on the estimated variance-covariance parameters of the four populations, listed in Table 4. The CHD and JPT populations are so close to each other that they can be distinguished only on eigenvector 3.

We estimated the variance-covariance parameters and the axes of variation for some of the HapMap [18] populations. An example is given in Figure 7, where the first three sample eigenvectors are plotted from the analysis of the four populations: Chinese in Denver (CHD), Gujarati Indians in Houston (GIH), Japanese in Tokyo (JPT), and Tuscan Italians (TSI) using markers on chromosome 1. The estimates of the variance-covariance parameters for these four populations are given in Table 4. The axes of variation calculated from these estimates are also shown in Figure 7 and were in consistent with the sample eigenvectors calculated directly from the raw variance-covariance matrix. It can be seen from Figure 7 that the two genetically very close populations, CHD and JPT, are contrasted only on the third eigenvector; the first two eigenvectors are used to address the difference between PHD+JPT vs GIH and TSI, and the difference between GIH vs TSI. We note that CHD and JPT can be distinguished not only by PCA together with other populations but also by PCA by themselves (data not shown). As shown in Table 4, the genetic diversity within GIH or TSI is so large that the average covariance between two random individuals both from GIH or from TSI is larger than the covariance between an individual from CHD and an individual from JPT. This explains why on eigenvector 3, where CHD and JPT are contrasted, the clusters of GIH and TSI are also elongated, showing a within-population structure.

**Table 4:** Estimates and the corresponding standard errors for the variance-covariance matrix of the four populations CHD, GIH, JPT, and TSI using HapMap data for chromosome 1.

	CHD	GIH	JPT	TSI
Sample size^a^	70	83	82	77
Variance	0.680(0.006)	0.661(0.008)	0.680(0.007)	0.665(0.008)
Covariance				
CHD	0.360(0.008)	0.279(0.008)	0.354(0.008)	0.260(0.007)
GIH		0.314(0.012)	0.280(0.008)	0.298(0.008)
JPT			0.357(0.008)	0.260(0.007)
TSI				0.330(0.008)

a Sample size: The number of individuals of each population.

### Application to genetic epidemiology

#### Correcting for population stratification using axes of variation

Now we turn to the issue of correcting for population stratification in genetic association studies. In the Eigenstrat method, the correction for stratification is performed by regressing out the variation caused by population structures [9]. In the theory of PCA, the random vector 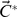 can be expanded as a linear combination of all PCs:
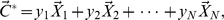
(39)where 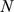 is the total number of individuals and
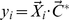
(40)is the 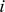
th PC. To correct for population stratification, we subtract the first 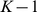 terms from this expansion, which are the variations due to the differences between the 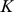 populations. The sum of the remaining terms, describing the variations between individuals,
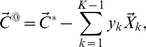
(41)is then used for disease association test.

The PCs removed for correcting population stratification in Equation (39) are those obtained using the “sample” markers in the Eigenstrat method and is referred to as sample PCs. Here, we propose to use the same strategy for correcting population stratification but with the PCs defined using the axes of variation (referred to as representative PCs or “population” PCs). Our motivation is as follows. When the Eigenstrat method is used, some of the genotype variations corresponding to within-population fluctuations in the sample eigenvectors are removed, in addition to those corresponding to population stratification. As shown in previous sections, these within-population fluctuations are mainly due to the finite “sample size” (i.e. limited number of markers) and thus are irrelevant to the issue of population stratification. Indeed, in practice, a proportion of the within-population fluctuations may be due to a subpopulation structure. However, as shown in previous sections, the fluctuations in the first few eigenvectors only partially represent this kind of subpopulation structure. Consider the example given in Figures 4 and 5. When the samples are thought of as coming from three distinct populations P1, P2+P3, and P4, only the first two eigenvectors should be used to correct for population stratification. The variation caused by the difference between P2 and P3 would then be partially removed; although the fluctuation described by 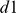 on eigenvector 1 would be taken into account, that described by 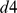 on eigenvector 3 would remain in the residual. So the variation caused by the difference between P2 and P3 would only be altered and not completely removed. Only partially removing the variations caused by a subpopulation structure may not be really helpful for reducing false-positive rates in a case-control study. In this specific example, where the subpopulation structure is the simplest, we could remove the corresponding variation by simply adding the third PC in the sum in Equation (41). In reality, however, subpopulation structures are far more complex and are hence represented by many PCs. Regressing out too many PCs in Equation (41) would remove too much inter-individual variation within a pure population and hence would significantly reduce the power of association tests. We therefore prefer to use the representative PCs in Equation (41) for removing the main variations caused by major population stratification, while keeping the variations due to subtle subpopulation structures unchanged. Since the “population” PCs are used here, the proposed method is referred to as popu-Eigenstrat.

Now let us derive the theoretical expression of the residuals in Equation (41). Since we use the representative PCs, for a given 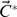, the vector 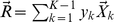, which is subtracted from 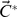, has a structure like
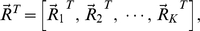
(42)and for each of the 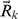,
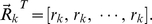
(43)In Text S3, it is shown that
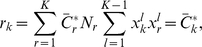
(44)where
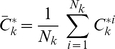
(45)is the mean of 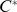 over individuals from population 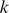. So it turns out that our representative PC-based correction is simply equivalent to subtracting the population group means for each individual. Our method does not even use any information about the variance-covariance parameters.

### Results of simulations

We conducted simulations for comparing the performance of the popu-Eigenstrat method with that of the original Eigenstrat method as well as with that of the covariate-adjustment method, which used population labels as a covariate. See Method section for details of the simulations. Table 5 shows the results of our simulations. For 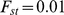, which is typical of differentiation between divergent European populations, the proposed method, popu-Eigenstrat, using the representative PCs, achieved almost the same rates of false-positive associations and comparable power as the original EIGENSTRAT method, which uses the sample PCs. Compared with the covariate-adjustment method, our method has slightly lower power, but also a lower rate of false-positive associations.

**Table 5:** Proportion of associations reported as significant (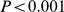) using different methods of stratification correction for simulated data.

		Without correction	Eigenstrat	popu-Eigenstrat	Covariate-adjustment
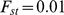					
StruInfo SNPs^a^	Causal^c^	0.6002	0.5129	0.5077	0.5651
	Null^d^	0.0281	0.0057	0.0052	0.0090
Specific SNPs^b^					
0.75∶0.30∶0.15	Causal^c^	0.0000	0.4106	0.4218	0.4690
	Null^d^	0.9938	0.0060	0.0059	0.0090
Specific SNPs^b^					
0.15∶0.30∶0.75	Causal^c^	1.0000	0.4105	0.3712	0.4253
	Null^d^	0.9730	0.0074	0.0073	0.0113
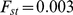					
StruInfo SNPs^a^	Causal^c^	0.6939	0.5263	0.5168	0.5738
	Null^d^	0.0144	0.0062	0.0068	0.0102
Specific SNPs^b^					
0.75∶0.30∶0.15	Causal^c^	0.0000	0.2175	0.4180	0.4674
	Null^d^	0.9947	0.0143	0.0058	0.0092
Specific SNPs^b^					
0.15∶0.30∶0.75	Causal^c^	1.000	0.5537	0.3572	0.4112
	Null^d^	0.9716	0.0130	0.0064	0.0094

a StruInfo SNPs: Allele frequencies were simulated based on the Balding-Nichols model and the SNPs were population structure informative.

b Specific SNPs: Allele frequencies of the populations were fixed to the specified values: 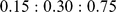 (proportional to the ratio of the sample sizes) or 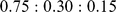 (inversely proportional to the ratio of the sample sizes).

c Causal: Genotypes were generated according to the proportion: 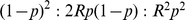 where 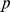 is the causal allele frequency and 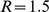 is the relative risk.

d Null: Genotypes were generated according to the Hardy-Weinberg proportion: 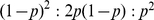. 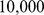 SNPs were simulated for each of the categories.

It is interesting to compare the results for the two different allele frequency sets for the Causal-Specific SNPs (see Methods for definitions of these simulated SNPs). When the allele frequency ratio was the inverse of the sample size ratio, the power was zero if no correction was performed. The reason was that in this case, even though a very high proportion of tests had very low p-values, the disease allele was incorrectly identified as the wild allele. The power increased to 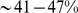 after the methods of stratification correction were applied. In contrast, when the allele frequency ratio was the same as the sample size ratio, the power without correction was as high as 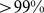
! Without population stratification, we could not have achieved such an extremely high power for the given sample size, allele frequencies and relative risk. In this case, the population with a high disease allele frequency was over-sampled and thus the difference of allele frequency between the case and control groups was enlarged. After the methods of stratification correction were applied, the power was reduced to its “normal” level (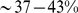). This observation indicates that special attention has to be paid to the population's allele frequency spectra when powers for stratification correction strategies are compared. For the causal-StruInfo SNPs in our simulation (see Methods for definitions of these simulated SNPs), some of the SNPs for which the allele frequency ratio was the inverse of the sample size ratio may have contributed to an increase in power after stratification correction was applied. However, more SNPs had allele frequency ratio with the same trend as the sample size ratio and hence contributed more to a reduction in power when stratification correction was applied.

For a smaller 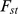, 0.003, we found that the performance of the original Eigenstrat method was poorer. The rate of false positives was reduced less by Eigenstrat method than by the other methods. The power was increased less by Eigenstrat method than it was by the other methods in the case when the ratio of disease-causing allele frequencies was the inverse of the ratio of sample sizes. Only when the two ratios were the same did the Eigenstrat method improve the power to significantly higher than the others. The other methods of stratification correction worked similarly as in the case of larger 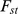. This can be explained as follows. As 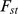 decreased, the distances between the populations decreased, and hence the fluctuations in the sample eigenvectors increased. This in turn made these sample PCs less representative of the population structure, resulting in a poorer performance in correcting for stratification.

We also examined how power was affected by regressing out too many PCs using Eigenstrat method. In the case of 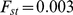, for the Causal-StruInfo SNPs, the power was reduced from 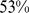 (see Table 5) obtained using only two PCs to 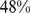, 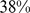 and 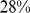 when using 50, 90 and 150 PCs, respectively.

Finally, the results on our comparison between Eigenstrat and popu-Eigenstrat should be taken with caution, because additional information (i.e. population memberships) was given to popu-Eigenstrat.

## Discussion

The Eigenstrat method is a powerful tool to detect and correct for population stratification by treating genotype data as “samples” of markers. In this work, we have provided a framework in which the large eigenvalues and the corresponding eigenvectors necessary for differentiating the population structure are theoretically connected to the variance-covariance parameters of the random vector of the allele frequencies of the populations. These variance-covariance parameters can serve as an alternative to the traditional 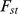 statistic for quantifying population relationships. In practice, our formulation provides theoretical guidance on how to correctly infer population structures from the pattern of the PC plot. Using the developed formulation, we have shown that there exists an asymptotic pattern on the PC plot as the sample size become large. We have also shown that the asymptotic pattern can be easily obtained by numerically solving the asymptotic form of the reduced eigen-equation for given covariance parameters and the relative sample sizes.

Based on our theoretical consideration and simulations, we have investigated the factors that affect the within-population fluctuations of the sample eigenvectors (as obtained from the Eigenstrat method) around the axes of variation. As the sample size becomes large, the overall asymptotic pattern of the PC plot quickly forms. The within-population fluctuations in the asymptotic pattern are then mainly determined by the number of markers and the subpopulation structure. These fluctuations corresponding to the very subtle subpopulation structures are entangled with the normal inter-individual variations within a pure population and hence can hardly be adjusted without significantly affecting the power of association tests.

These conclusions led us to a novel method of correcting for population stratification: We can regress out the representative PCs, instead of the sample PCs as done in Eigenstrat theory. We theoretically showed that this method is equivalent to simply removing the population mean of the allele counts. Therefore, implementation of the proposed method becomes trivial, whence the samples' population memberships are known (either self-reported or identified using the Eigenstrat method or any other methods, such as STRUCTURE). Our simulation studies showed that the proposed method worked as well as the Eigenstrat method for reducing false positive-rates and for maintaining the power of association tests. The proposed method outperformed the method of simply using the population label as a covariate in reducing false-positive rates, and it had slightly lower power. Our proposed method can also be used in candidate gene association studies or replication studies as long as the population memberships are known and a trend test is preferred.

In the present work, we have not considered admixture of populations. As shown in [8], PCA carried out on samples that include admixed individuals produces an interesting pattern on the PC scatter plot: the admixed samples are lying along a line between the two source populations. Similar patterns have been observed for other populations [13], [16]. Work is currently in progress to extend our theoretical formulation to the situation of admixture in order to explain the observed patterns.

## Methods

### Simulations of population structure

Following [4] and [9], we simulated genotype data for a specified number of populations with specified values of 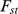 using the Balding-Nichols model [21]. The ancestral allele frequency 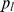 was first generated from the uniform distribution on 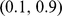 for each locus 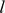. The allele frequencies in population 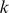 were then drawn from a beta distribution with parameters 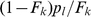 and 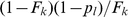, where 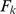 is the 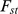 for population 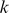. No linkage disequilibrium was considered here. Distances between a pair of population was determined by the populations' 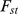
s with the ancestral population. Only when 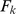 is chosen to be the same for all populations to be simulated does it become an estimate of 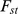 for all the populations.

### Simulations of association tests

Our simulations for association tests were similar to those reported in [9]. One of the differences between our simulations and those in [9] is that we considered three populations rather than two populations. In our simulations, we assumed that the prior probability of sampling individuals from each of these three populations was the same, 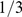. We assumed that the ratio of disease prevalences in the three populations was 1∶2∶5, and that these prevalences were very small. The numbers of cases and controls simulated for each population were their expected values, namely, 30, 60, 150 for cases and 80, 80, 80 for controls.

For each individual in each population, we simulated four different categories of SNPs. The first and second categories were generated using allele frequencies based on the Balding-Nichols model [21] with 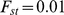 or 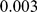 for all populations, and the SNPs were thus population-structure-informative (StruInfo SNPs). The genotype data were generated differently for the first and second categories. For the first category, data for both cases and controls were generated in the same way, by using Hardy-Weinberg equilibrium, and hence were not associated with the disease. SNPs in the first category were thus referred to as Null-StruInfo SNPs. We simulated genotypes of 10,000 Null-StruInfo SNPs. They were first used to infer the variance-covariance matrix of the three populations and the sample PCs and then served as replicates for estimating the type I error rate. For the second category of SNPs, referred to as Causal-StruInfo SNPs, the genotypes were simulated differently for cases and controls. For controls, the simulation of genotypes was the same as for the Null-StruInfo SNPs, whereas for cases, we used a risk model with a relative risk 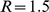 for the causal allele. A case individual was assigned genotype 0,1, or 2 with probabilities 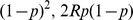, or 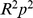, respectively, where 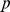 is the causal allele frequency.

We also simulated 10,000 SNPs for each of the third and fourth categories. The third category of SNPs, referred to as Null-Specific SNPs, were disease-independent, like the Null-StruInfo SNPs, but had a fixed allele frequency set for the three populations. For the fourth category, Causal-Specific SNPs, the allele frequencies for the three populations were also fixed, but the cases and controls were simulated differently, as for the Causal-StruInfo SNPs, using the same relative risk, 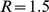. We used two different allele frequency sets for the specific SNPs: 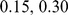, and 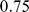; and 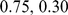 and 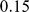. In the first set, the ratio of the allele frequencies of the populations was the same as that of the sample sizes. In the second set, the ratio of the allele frequencies was the inverse of that of the sample sizes. The Null-Specific SNPs were intended for estimating type I error rate and the Causal-Specific SNPs for estimating power for a specific allele frequency set in the populations.

Following [8], we used the Armitage trend 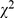 statistic for association tests without stratification correction (without-correction), and we used the generalized Armitage trend 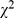 statistic when stratification was corrected for by regressing out the sample PCs (Eigenstrat) or the representative PCs (popu-Eigenstrat). For a fourth test, we used the population label as a covariate (covariate-adjustment). Association statistics producing a P value 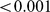 were reported as significant.

Programs and scripts used in this work are available at https://cge.mdanderson.org/dma/User/ProgramsScripts/popuPCA/: (a) VarCov, a C++ program for calculating the sample variance-covariance matrix from genotype data; (b) EstimateSigma, a C++ program for estimating the variance-covariance parameters from the sample variance-covariance matrix; (c) ReducedMat.awk, an awk script for calculating the reduced matrix from the variance-covariance parameters and the sample sizes; and (d) An R script for calculating the eigenvalues and eigenvectors of the reduced matrix.

## Supporting Information

Text S1 Derivation of Equations (8–10)
(0.03 MB PDF)

Text S2 Derivation of Equation (28) and Equation (34)
(0.03 MB PDF)

Text S3 Derivation of Equation (44)
(0.02 MB PDF)
