# Supplementary material for: Theoretical Formulation of Principal Components Analysis to Detect and Correct for Population Stratification
Source: PLoS One. 2010 Sep 17;5(9):e12510. doi: 10.1371/journal.pone.0012510 (PMC2941459; doi:10.1371/journal.pone.0012510)
Supplement: Text S1 — Derivation of Equations (8–10) (0.03 MB PDF) [file pone.0012510.s001.pdf]

Text S1: Derivation of Equations (8-10)

Without loss of generality, suppose that there are two populations. For a random marker, the allele frequencies for these two populations are p_1 and p_2. Denote the variance-covariance matrix of p_1 and p_2 by

$$V_F = \begin{pmatrix} \Sigma_1^2 & \Sigma_{12} \\ \Sigma_{12} & \Sigma_2^2 \end{pmatrix}.$$

Suppose that individuals $1, 2, \cdots, N_1$ are from population 1 and individuals $N_1+1, N_1+2, \cdots, N$ are from population 2. For an individual from population 1, say individual 1, we can write the marginal probability of variant allele count C_1 as

$$\begin{aligned}
P(C_1) &= \sum_{C_2,\cdots,C_N} \int \mathrm{d}p_1 \mathrm{d}p_2 P(C_1, C_2, \cdots, C_N | p_1, p_2) P(p_1, p_2) \\
&= \sum_{C_2,\cdots,C_N} \int \mathrm{d}p_1 \mathrm{d}p_2 P(C_1|p_1) P(C_2, \cdots, C_N | p_1, p_2) P(p_1, p_2) \\
&= \int \mathrm{d}p_1 P(C_1|p_1) \int \mathrm{d}p_2 P(p_1, p_2) \left[\sum_{C_2} P(C_2|p_1)\right]^{N_1-1} \left[\sum_{C_N} P(C_N|p_2)\right]^{N-N_1} \\
&= \int \mathrm{d}p_1 P(C_1|p_1) \int \mathrm{d}p_2 P(p_1, p_2).
\end{aligned}$$

Similarly, we have, for an individual in population 2, say, C_N,

$$P(C_N) = \int \mathrm{d}p_2 P(C_N|p_2) \int \mathrm{d}p_1 P(p_1, p_2),$$

and their joint marginal probability is

$$P(C_1, C_N) = \int \mathrm{d}p_1 \mathrm{d}p_2 P(p_1, p_2) P(C_1|p_1) P(C_N|p_2).$$

For two individuals in the same population, say C_1 and C_2,

$$P(C_1, C_2) = \int \mathrm{d}p_1 P(C_1|p_1) P(C_2|p_2) \int \mathrm{d}p_2 P(p_1, p_2).$$

Using these marginal probabilities and the Hardy-Weinberg proportion, we can prove that

$$\begin{aligned}
\bar{C_1} &= \sum_{C_1} C_1 P(C_1) C_1 = \int \mathrm{d}p_1 \int \mathrm{d}p_2 P(p_1, p_2) \sum_{C_1} C_1 P(C_1|p_1) \\
&= \int \mathrm{d}p_1 P(p_1) \left[2p_1^2 + 2p_1(1-p_1)\right] = 2\bar{p_1},
\end{aligned}$$

and similarly

$$\bar{C_1^2} = 2\bar{p_1} + 2\bar{p_1^2},$$

and hence

$$\mathrm{VAR}(C_1) = 2\Sigma_1^2 + 2\bar{p_1}(1-\bar{p_1}),$$

which is Equation (8) in the main text. Equations (9) and (10) in the main text can be similarly proven.
