# Supplementary material for: Theoretical Formulation of Principal Components Analysis to Detect and Correct for Population Stratification
Source: PLoS One. 2010 Sep 17;5(9):e12510. doi: 10.1371/journal.pone.0012510 (PMC2941459; doi:10.1371/journal.pone.0012510)
Supplement: Text S2 — Derivation of Equation (28) and Equation (34) (0.03 MB PDF) [file pone.0012510.s002.pdf]

Text S2: Derivation of Equation (28) and Equation (34)

For any off-diagonal element of the matrix in Equation (27), we can rewrite $\sigma'_{kk'}$ as a sum of these two parts:

$$\sigma'_{kk'} = \hat{\sigma}_{kk'} + \tilde{\sigma}_{kk'},$$

where $\hat{\sigma}_{kk'}$ and $\tilde{\sigma}_{kk'}$ are given in Equations (35) and (36), respectively, each of which is homogeneous in terms of the sample sizes $(N_1, N_2, \cdots, N_K)$. Similarly, the kth diagonal term of the matrix in Equation (27) can be rewritten as

$$\sigma'^2_k + (N_k - 1)\sigma'_{kk} = \sigma^s_k + N_k \sigma'_{kk} = H_k + S_k,$$

with

$$H_k = N_k \left(\sigma_{kk} + \frac{1}{N^2} \sum_{m,n=1}^{K} N_m N_n \sigma_{mn} - \frac{2}{N} \sum_{m=1}^{K} N_m \sigma_{km} \right)$$

and

$$S_k = \frac{N_k}{N^2} \sum_{m=1}^{K} N_m \sigma^s_m - \frac{2N_K}{N} \sigma^s_k + \sigma^s_k.$$

From these expressions, we have, after some algebra,

$$H_k = -\sum_{k' \neq k}^{K} N_{k'} \hat{\sigma}_{kk'}$$

and

$$*S_k = -\sum_{k' \neq k}^{K} N_{k'} \tilde{\sigma}_{kk'},$$

which in turn leads to Equation (28).

Note that the behaviors of the two parts of $\sigma_{kk'}$ are different when $N \to \infty$:

$$\hat{\sigma}_{kk'} \sim O(1),$$

whereas

$$*\tilde{\sigma}_{kk'} \sim O\left(\frac{1}{N}\right).$$

Ignoring the second part for all elements, $\tilde{\sigma}_{kk'}$, in Equation (28) leads to the asymptotic form of the reduced eigen-equation, (34).
