# Supplementary material for: Theoretical Formulation of Principal Components Analysis to Detect and Correct for Population Stratification
Source: PLoS One. 2010 Sep 17;5(9):e12510. doi: 10.1371/journal.pone.0012510 (PMC2941459; doi:10.1371/journal.pone.0012510)
Supplement: Text S3 — Derivation of Equation (44) (0.02 MB PDF) [file pone.0012510.s003.pdf]

Text S3: Derivation of Equation (44)

Here, we calculate the vector $\vec{R}$, which is to be subtracted from the original genotype vector, as defined in Equation (42). For the component of $\vec{R}$ corresponding to the kth population,

$$r_k = \sum_{m=1}^{K-1} x_k^m y_m = \sum_{m=1}^{K-1} x_k^m \sum_{l=1}^{K} \bar{C}_l^* N_l x_l^m.$$

Using

$$\sum_{l=1}^{K} \bar{C}_l^* N_l \equiv 0$$

and the fact that

$$x_l^K = x^K$$

does not depend on l (corresponding to the only zero eigenvalue), we have

$$r_k = \sum_{m=1}^{K-1} x_k^m \sum_{l=1}^{K} \bar{C}_l^* N_l x_l^m + x_k^K \sum_{l=1}^{K} \bar{C}_l^* N_l x_l^K = \sum_{l=1}^{K} \bar{C}_l^* N_l \sum_{m=1}^{K} x_k^m x_l^m.$$

Using results given in [19] (pp.467-470), it can be shown that

$$\sum_{m=1}^{K} x_k^m x_l^m = \frac{1}{N_k} \delta_{kl},$$

where

$$\delta_{kl} = \begin{cases} 1 & (k = l) \\ 0 & (k \neq l). \end{cases}$$

Substituting Equation (70) into Equation (60) yields Equation (44).
